# The first complete assembly for a lungless urodelan with a “miniaturized” genome, the Northern Dusky Salamander (Plethodontidae: *Desmognathus fuscus*)

**DOI:** 10.1093/g3journal/jkaf157

**Published:** 2025-07-07

**Authors:** Edward A Myers, Alexander A Stewart, Aidan E O’Brien, Jeramiah J Smith, R Alexander Pyron

**Affiliations:** Department of Herpetology, California Academy of Sciences, San Francisco, CA 94118, United States; Department of Vertebrate Zoology, Smithsonian National Museum of Natural History, Smithsonian Institution, Washington, DC 20560, United States; Department of Ecology and Evolutionary Biology, The University of Arizona, Tucson, AZ 85721, United States; Department of Biology, University of Miami, Coral Gables, FL 33124, United States; Department of Biology, University of Kentucky, Lexington, KY 40508, United States; Department of Vertebrate Zoology, Smithsonian National Museum of Natural History, Smithsonian Institution, Washington, DC 20560, United States; Department of Biological Sciences, The George Washington University, Washington, DC 20052, United States

**Keywords:** amphibia, urodela, plethodontidae, *Desmognathus fuscus*, genome, transcriptome, transposable elements, genome assembly

## Abstract

Salamanders have some of the largest genomes among animals, driven primarily by expansion of repeat elements and slow rates of DNA loss. However, species in the lungless genus *Desmognathus* (Plethodontidae) have some of the smallest genomes at ∼13 to 15 GB, though previous sequencing attempts with short-read assemblies were still fragmentary. Here, we assemble an annotated draft genome of *Desmognathus fuscus* using PacBio HiFi reads and transcriptomic data from 2 specimens from the same population. The combined dataset resulted in a 16.1 GB assembly in 19,632 contigs and an L/N50 of 2,459/1.74 MB, with the longest contig at 27.9 MB. This may still be slightly larger than the true size due to incomplete resolution of repeat regions. The assembly is highly complete with 93% of the 5,310 Tetrapoda BUSCO orthologs. Only 25% of the genome is single copy with 75% corresponding to transposable elements, mostly LTRs (36% of the genome) and LINEs (15%). We estimate an early expansion and slow contraction of LINEs followed by a quick recent expansion of both LTRs and DNA transposons. We identified 12,408 protein-coding genes with a common name in 1 or more reference databases. Finally, we used mother-offspring RNAseq data from an unrelated *D. fuscus* clutch to create a high-density linkage map, yielding 14 primary linkage groups and 2 smaller scaffolds covering 7.5 Gb and incorporating 46.5% of the genome and 60% of the mRNAs. This chromosome-scale assembly represents a new benchmark for plethodontid salamander genomics.

## Introduction

Salamanders have some of the largest genomes known in animals, ranging from ∼8 to 130 GB (Sclavi and Herrick 2019). They are model systems for numerous topics including the genetic basis of evolutionary links between life history and genome size (Lertzman-Lepofsky *et al.* 2019; Mueller *et al.* 2023), as well as aging, vision, and immunity (Roth *et al*. 1997; Joven *et al.* 2019; Bolaños-Castro *et al.* 2021). In contrast, their gigantic genomes have frustrated attempts at contiguous assembly (Pyron *et al.* 2024), given the difficulties of mapping extensive repeat landscapes with short-read data. The first species with a complete assembly was the axolotl (Ambystomatidae: *Ambystoma mexicanum*; Keinath *et al.* 2015; Evans *et al.* 2018; Nowoshilow *et al.* 2018; Smith *et al.* 2019), a common lab model yielding insight into pertinent questions in gene regulation, transcriptional control, and regeneration (Schloissnig *et al.* 2021). Up to 19 GB (∼60%) of the 32 GB genome may consist of repetitive sequence (Keinath *et al.* 2015), resulting in the giant size. Another genome for a newt (Salamandridae: *Pleurodeles waltl*) comprises a 20 GB chromosome-scale assembly providing extensive insight into regeneration and repeat-element diversity (Elewa *et al.* 2017; Brown *et al.* 2025). Finally, a 23 GB unscaffolded draft for a second newt (*Calotriton arnoldi*)—pooling reads from 2 siblings—yielded a contig-level assembly with ∼82% of the BUSCO Vertebrata loci (Talavera *et al.* 2024).

Within salamanders, significant variation in genome size is observed in the lungless family Plethodontidae (∼14 to 76 pg; Sessions and Larson 1987), with a major evolutionary decrease in the eastern Nearctic genus *Desmognathus* (∼13 to 15 GB; Liedtke *et al.* 2018). Genome size changes in salamanders are associated with ecological constraints and life-history strategies (Lertzman-Lepofsky *et al.* 2019), and *Desmognathus* notably represent a reversion to bi-phasic lifestyle with metamorphosing aquatic larvae (Chippindale *et al.* 2004). Consequently, the *Desmognathus* genome is of exceptional interest both for the broad applicability of salamanders as a model system for traits such as regeneration (Sessions and Wake 2021), as well as the evolutionary linkage between ecological adaptations, repeat elements, and genome size (Jockusch 1997; Sun *et al.* 2012a, 2012b; Itgen *et al.* 2022; Mueller *et al.* 2023).

Previous studies using a short-read shotgun-sequencing approach to generate partial genomic coverage in *Desmognathus* and relatives showed that up to 30% of the genome consists of long terminal repeat (LTR) retrotransposons, primarily Ty3 elements representing up to 20% of the reads for each species (Sun *et al.* 2012a, 2012b). Additionally, these species show extremely slow rates of DNA loss (<0.05 bp/substitution) within these elements, suggesting a primary mechanism for genome expansion (Sun *et al.* 2012a, 2012b). Those authors also noted that their results for LTR content were likely substantial underestimates, emphasizing the need for more complete and contiguous assemblies to characterize the repeat-element landscape in plethodontids and other salamanders (Sun and Mueller 2014).

While single-individual genomes remain the gold standard (Rhie *et al.* 2021), pooled-sample approaches may be necessary for draft assemblies of very small organisms that may not yield sufficient DNA for high-coverage long-read sequencing (Goldberg *et al.* 2024), particularly if one desires long-range data such as Hi-C chromatin conformation capture. On the one hand, erythrocyte nucleation in salamanders (see Mueller *et al.* 2008 and references therein) provides for easier purification of high molecular weight DNA, facilitating longer read lengths from smaller amounts of blood and tissue. On the other hand, many plethodontids are very small—as little as ∼25 mm SVL (Parra-Olea *et al.* 2016)—making it difficult to recover sufficient input material for high-coverage high-contiguity assemblies from a single individual.

Here, we use HiFi reads from the Pacific Biosciences Revio platform to generate an approximately complete assembly (∼16 GB) with high coverage (∼24×) for the Northern Dusky Salamander (Plethodontidae: *Desmognathus fuscus*), from blood and liver collected from 2 individuals from the same population. Annotation of genes and repeat elements showed that ∼75% of the genome is repetitive, and as little as ∼25% of the genome is single copy (in line with previous estimates; Morescalchi and Olmo 1982), identifying ∼12 k protein-coding genes. We then used mother-offspring RNAseq data from a separate clutch to create a high-density linkage map and scaffold the contigs to a chromosome-level assembly of 1N = 14, with the longest chromosomal linkage group (LG) ∼2.8 GB. Extensive opportunities remain for annotated chromosome-level assemblies of *Desmognathus,* plethodontid relatives, and other salamander families to understand the ecological and functional basis of genome-size evolution. In the interim, this assembly for *D. fuscus* provides an initial reference for future studies of comparative genomic analysis in lungless salamanders.

## Materials and methods

### Sampling and sequencing

**Fig. 1.**
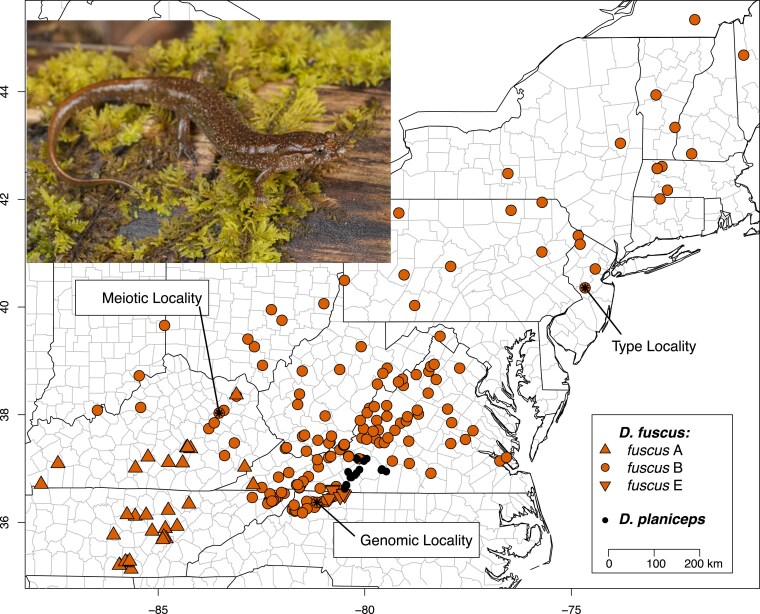
Distribution map of *D. fuscus* and its sister species *D. planiceps* based on recent taxonomic revisions (Pyron and Beamer 2023a), indicating the type locality of the former (Green 1818; Pyron and Beamer 2020; Dubois *et al.* 2022), and the sites in North Carolina and Kentucky where AMNH A-194068–9 (genomic locality) and the mother-offspring clutch (meiotic locality) were sampled, respectively. This species contains phylogeographic sublineages A, B, and E (indicated by marker shape) which may prove to be distinct species in the future. The B lineage sampled here is nominotypical. Inset photo shows a typical individual in life from the genomic locality (photo courtesy of Max A. Seldes, GW).

Collecting and research were conducted under NCWRC and KDFW permits and GW and UKY IACUC animal-use protocols. One of us (R.A.P.) collected 2 *D. fuscus* (RAP3214–5) from the “*fuscus* B3' lineage (Pyron *et al.* 2022; Pyron and Beamer 2023a) on 20 May 2023 in Bell Branch, a small creek on the Thurmond Chatham Game Land in Wilkes Co., North Carolina (Fig. 1). The specimens were a relatively small subadult (∼32 mm SVL) and adult male (∼43 mm), captured within ∼3 to 4 m of each other under rocks and logs on the stream bank. Vouchers were deposited at the American Museum of Natural History (AMNH A-194068–9).

Following euthanasia, we preserved blood and liver from both specimens and kidney and associated ducts from AMNH A-194069 only. We used an NEB Monarch HMW DNA Extraction Kit for Cells & Blood (T3050S) with minimal shearing or fragmentation to purify ultra-high molecular weight genomic DNA from AMNH A-190468. Initial QC at the University of Maryland Institute for Genome Sciences (IGS; http://www.marylandgenomics.org/) using an Agilent Femto Pulse system showed 6,319 ng of DNA at 71 ng/µL in 89 µL of buffered extract (average size 80 kb, 85% ≥ 30 kb), which was sufficient for 2 PacBio Revio SMRT Cells. We subsequently provided flash-frozen liver tissue from AMNH A-194069 to the sequencing center for additional UHMW DNA extraction. This yielded 22,947 ng of DNA at 81 ng/µL in 284 µL, with an average size of 103 kb and 79% > 30 kb. For each specimen, the IGS performed 1 PacBio HiFi Library Preparation with standard size selection of 15 to 18 kb, which supplied input libraries for 2 PacBio Revio SMRT Cell 25 M Sequencing runs (HiFi/CCS mode, 24-h movie).

For AMNH A-194068, the 2 cells yielded 88.3/85.3 Gb from 6.03/5.96 million CCS reads, respectively. This represents a total of ∼174 GB of data representing ∼12× total coverage of the expected ∼15 GB *Desmognathus* genome (Liedtke *et al.* 2018). The read N50 was 135,250/117,250 bp for polymerase reads and 14,754/14,413 bp for CCS reads, with a range of 107 to 57,921 bp for the latter. For AMNH A-194069, the 2 cells yielded 99.1/92.6 gigabases from 5.47/5.02 million CCS reads, respectively for a total of ∼192 GB of data representing ∼13× coverage. These are slightly better results than for the smaller specimen, possibly representing a difference in the extraction quality or tissue type. The read N50 was 124,250/113,250 bp for polymerase and 19,606/16,869 bp for CCS, with a range of 49 to 60,229 bp for the latter.

Additionally, we sequenced total RNA transcriptomes from liver (AMNH A-194068) and kidney (AMNH A-194069) at Psomagen Inc. (Rockville, MD) using the TruSeq Stranded Total RNA with Ribo-Zero Gold Human/Mouse/Rat kit. For each sample, we sequenced ∼40 M 150 bp paired-end reads on a NovaSeq6000 S4 flow cell. We used fastQC (Brown *et al.* 2017) as an initial quality-control screen on the raw Illumina reads, which were uniformly high quality, and filtered and trimmed them with Trimmomatic (Bolger *et al.* 2014).

Concomitantly, A.A.S. and A.E.O. collected a nest-guarding female *D. fuscus* and 21 embryos on 28 August 2021 from Leatherwood Creek in Menifee Co., Kentucky, part of the “*fuscus* B1/B2” lineage (Pyron *et al.* 2022). The embryos were late in development with complete pigmentation, fully developed gills, and feet with distinct digits. The female and her clutch were euthanized, and the embryos were dechorionated using fine-tip forceps. Dissected adult tissues and dechorionated embryos were placed in 1.7 mL tubes, flash frozen in liquid nitrogen, and stored at −80 °C. RNA was extracted from 4 maternal tissues (brain, liver, ovaries + kidney, and tail tip) and from whole embryos using standard TRIzol extraction.

**Fig. 2.**
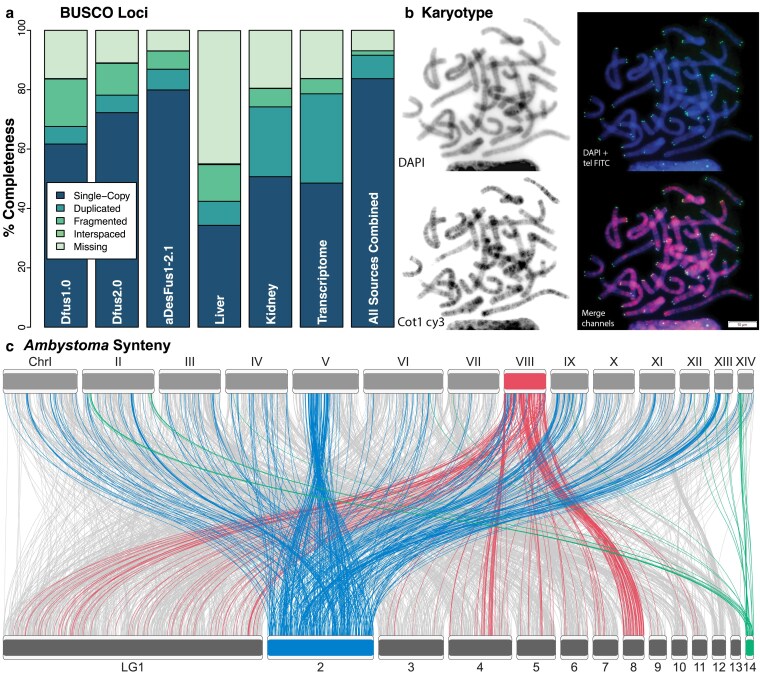
a) BUSCO results from compleasm for the various genome and transcriptome assemblies compared to the 5,310 Tetrapoda orthologs. b) Karyotype from the meiotic locality confirming 2*n* = 28, highlighting telomeres (in green) and repeat regions (in black). Dyes, probes, and stains for visualization are DAPI (for blue fluorescence of DNA), telomeric FITC (for green fluorescence of telomeres), and C_0_*t1* (for repetitive elements) labeled with Cy3 (for orange/red fluorescence), with merged channels highlighting highly repetitive regions. c) Synteny plot of the *Ambystoma* genome GCA_002915635.3 (Schloissnig *et al.* 2021; top) mapped to the scaffolded LG of the aDesFus1-2.1 assembly (bottom) for 3,302 shared Tetrapoda BUSCO loci identified for both in compleasm. We observe modest conservation, highlighting the similarities of chromosomes and LG8 and LG14, and a large, conserved region that is inverted between chromosome 5 and LG2. In general, we observed exceptional dispersal of genomic elements across the ∼158Ma divergence between the 2 genomes.

The extracted RNA was assessed for quality on a bioanalyzer (Agilent 2100 Bioanalyzer; Agilent RNA 6000 Nano Kit) and via 1% agarose gel electrophoresis. Samples were sent to Novogene Corporation Inc. (Davis, CA, USA) for sequencing. Directional mRNA libraries were prepared using the NEBNext Ultra II Directional RNA Library Prep Kit for Illumina and sequenced on a single lane of an Illumina NovaSeq 6000. We generated ∼7 GB of 150 bp paired-end Illumina RNAseq from 20 embryos and 4 maternal tissues (brain, liver, ovaries + kidney, and tail tip). We also generated chromosomal spreads and hybridization signals from a second individual from the same population using standard methods (Keinath *et al.* 2015) for generating spreads from large salamander chromosomes for visualization to confirm 2*n* = 28. Chromosomes were hybridized with labeled C_0_*t* DNA (C_0_ representing the initial concentration of DNA and *t* the time in seconds to renature) and a pan-telomere peptide nucleic acid probe (Fig. 2).

### Assembly and scaffolding

We assembled the HiFi reads using *hifiasm* 0.19.8-r603 (Cheng *et al.* 2021). Given the low coverage of the individual specimens, we altered the default settings by setting the expected haploid genome size to 14 GB (‘−hg-size 14g’) and the minimum histogram count for estimating *k-*mer coverage to 2 (‘−min-hist-count 2'). Under these settings, we estimated a diploid assembly for each specimen separately. Individual coverage was not high enough (∼12×) for well-resolved and contiguous phased haplotypes (initial results were highly fragmented), so we instead present the haplotype-merged primary assembly (‘−primary’) for each individual.

Increasing coverage has been shown to result in dramatically higher-quality assemblies up to a threshold of ∼30×, at least in some species with smaller (∼1.6 GB) genomes (Xie *et al.* 2023). Accordingly, we merged the reads from the 2 individual specimens (∼366 GB total reads) for a higher-coverage (∼24×) assembly, treating the genome as polyploid (‘−n-hap 4') to account for the possibly divergent or heterozygous haplotypes across the 2 individuals. As the assemblies appeared to contain excess contigs from incomplete resolution of highly repetitive regions, we used 2 rounds of *purge_dups* to eliminate overlaps (Guan *et al.* 2020).

For the transcriptome, we analyzed the reads from both individuals (kidney for AMNH A-194068 and liver from AMNH A-194069) individually in *Trinity* 2.15.1 (Grabherr *et al.* 2011) to assemble transcript sequences as well as combining them for a consensus assembly. After assembly, we filtered the transcripts for quality and residual adapter sequence using *bbduk* in the *bbmap* package (https://sourceforge.net/projects/bbmap/).

We assessed initial gene completeness on the genome and transcriptome assemblies using *compleasm* 0.2.5 (Huang and Li 2023) using the *tetrapoda_odb10* reference database of 5,310 loci (Manni *et al.* 2021a, 2021b). Finally, we extracted, assembled, and annotated the mitochondrial genomes from the individual assemblies using the *MitoHiFi* pipeline (Uliano-Silva *et al.* 2023) with the *MitoFinder* annotation tool (Allio *et al.* 2020) and *ARWEN* for tRNA detection (Laslett and Canbäck 2008). Analyses were performed on the SI High Performance Computing Cluster “Hydra” (https://doi.org/10.25572/SIHPC), the GW HPCC Pegasus Cluster (MacLachlan *et al.* 2020), and the UK Morgan Compute Cluster.

We then performed chromosomal scaffolding using an RNAseq linkage map generated for the Kentucky specimens. While this population is from the same species (*D. fuscus*), it does represent a different phylogeographic sublineage (B1/2 vs B3; see Pyron *et al.* 2022), for which the level of genomic divergence may have affected the accuracy and success of contig placemetn. Future comparison to other strategies such as Hi-C scaffolding from more closely related populations may be illustrative and result in more comprehensive assemblies.

We generated the linkage map using SNPs segregating in a clutch of 19 embryos that were collected with a nest-guarding female (presumed parent). We generated genotypes for the female and 19 offspring using RNAseq data from the whole embryos and 4 maternal tissues. We assembled these sequences using *Trinity* (Grabherr *et al.* 2011; Haas *et al.* 2013) and the *filter_low_expr_transcripts.pl* script from the *Trinity-RNAseq* package to define a representative (primary) transcript for each locus. We called SNPs by realigning maternal and embryo reads to this set of representative transcripts using *BWA* (Li and Durbin 2009) and processed alignments with *BCFtools*. To generate a framework map, we used sites that (i) were heterozygous for the female, (ii) that segregated heterozygous vs a single homozygous genotype among her offspring (presumably homozygous for the same genotype across all sites), and (iii) yielded genotypes that were replicated 5 or more times in the dataset.

Next, we performed linkage analysis in *JoinMap5* using analysis methods for the F1 outbred crossing design (Stam 1993). We constructed a high-confidence linkage map using a subset of markers with highly replicated genotypes (those wherein the same segregation patterns across all 20 offspring were observed 5 or more times). Additional markers were added to this map if >80% of individuals had identical genotypes to framework markers when considering segregation in both coupling and repulsion phases. We then used this map as a framework to scaffold the assembled contigs using *Allmaps* (JCVI utility libraries 1.4.2; Tang *et al.* 2015), with weights for each position scaled to the number of genotype mismatches relative to a framework marker (mismatches/weight: 0/20, 1/8, 2/4, 3/2, 4/1). We evaluated the resulting scaffolds using the *geneticmap.py* “ld” function of *JCVI utility libraries* 1.4.2 and manually joined scaffolds showing high degrees of association at their tips. We then calculated synteny with *Ambystoma mexicanum* (GCA_002915635.3) using the BUSCO loci identified by *compleasm* (Fig. 2).

Finally, we annotated the scaffolded assembly for transposable elements and functional genes using 2 primary approaches. We used the *EarlGrey* v4.4.2 pipeline (Baril *et al.* 2024) to identify and annotate transposable elements using the default options including a 100 bp minimum length and 10 iterations of BLAST, extract, extend. We then used the *funannotate* pipeline (Palmer and Stajich 2020) for functional genes, including the train, predict, update, and annotate steps. For this, we included the RNAseq data from the 2 genomic samples (liver and kidney) and the 4 tissues (brain, liver, ovaries + kidney, and tail tip) from the female parental in the linkage analysis as evidence. We used the Tetrapoda BUSCO database with *Xenopus tropicalis* as the seed species, set the maximum intron length to 20 kb and maximum repeat length to 6 kb based on *Ambystoma* (Smith *et al.* 2019), incorporated masked repeats in *EVidenceModeler* (Haas *et al.* 2008), and used both *eggNOG-mapper* v2 (Cantalapiedra *et al.* 2021) and *InterProScan* 5 (Jones *et al.* 2014) in the annotate step.

## Results and discussion

For AMNH A-194068, the 2 partially phased haplotypes were 15.5/13.7 GB, and the primary assembly was 19.0 GB, likely reflecting incomplete resolution of highly repetitive regions given the lower coverage. A single round of *purge_dups* yielded a purged primary assembly of 15.9 GB in 51,453 contigs with an L/N50 of 10,433/466 Kb and an L/N90 of 1,518/1.02 MB. The longest contig is 6.87 MB, with 1,619 contigs >1 Mb, and 45,297 > 50 Kb, representing 98.7% of the genome. Similarly, for AMNH A-194069, the 2 partially phased haplotypes were 16.2/14.2 GB, and the primary assembly was 19.2 GB. A single round of *purge_dups* yielded a purged primary assembly of 15.8 GB in 29,360 contigs with an L/N50 of 5,466/864 Kb and an L/N90 of 774/1.97 MB. The longest contig is 12.5 MB, with 4,215 contigs >1 Mb, and 27,385 > 50 Kb, representing 99.6% of the genome. We named these assemblies *Dfus1.0* and *Dfus2.0* (‘see *Data Availability Statement*’).

The initial merged run yielded a primary assembly of 24.0 GB in 64,970 contigs with an L/N50 of 5,301/1.0 MB, an L/N90 of 279/5.1 MB, suggesting both a dramatic increase in contiguity with the greater sequencing depth (∼24× over ∼12×), and a substantial amount of duplicated haplotigs (∼9 GB) in the “polyploid” assembly. Two rounds of *purge_dups* yielded a purged primary assembly of 16.1 GB in 19,632 contigs with an L/N50 of 2,459/1.74 MB and an L/N90 of 282/5.0 MB. The longest contig is 27.9 MB, with 4,924 > 1 Mb, and 17,080 > 50 Kb, representing 99.5% of the genome. This assembly is still roughly 1 order of magnitude less contiguous than other recent urodelan genomes such as *Pleurodeles* (N50 ∼ 45.6 MB; Brown *et al.* 2025), potentially attributable to both lower coverage (∼24× here vs ∼41× in the newt) and artifacts from pooling samples. Adapting the naming rules from the Vertebrate Genome Project (Rhie *et al.* 2021), we labeled this *aDesFus1-2.1*—indicating pooling of the first 2 individuals—and treat it as our primary assembly for *D. fuscus* (Fig. 2).

A previous study sampling 4 species spanning the range of sizes, ecotypes, and phylogeny in *Desmognathus* (*Desmognathus amphileucus, Desmognathus monticola, Desmognathus perlapsus,* and *Desmognathus wrighti*) showed a uniform diploid karyotype of 2*n* = 28, suggesting that this structure is conserved across the genus (Hally *et al.* 1986), and we confirm this here for *D. fuscus* (Fig. 2b). While most authors have reported *C-*values of 14 to 16 pg for *Desmognathus* species (Hally *et al.* 1986; Sessions and Larson 1987; Liedtke *et al.* 2018), some studies have reported values up to 18 to 22 pg for species including *D. fuscus* (Bachmann 1970; Olmo 1974). Methods for determining genome size vary among these studies (e.g. Feulgen densitometry, flow cytometry), potentially explaining some of the variation. As the taxonomy of *Desmognathus* and *D. fuscus* in particular has shifted dramatically in recent years (Pyron and Beamer 2023a), taxonomic comparisons to previous results are difficult since sampling localities were often not reported for measured specimens. The assembly here may be slightly larger than reality (i.e. a true size of ∼14 to 15 GB) due to incomplete resolution of repeat regions, or there may be substantial variation in genome size across the genus or even within species. Future assemblies with telomere-to-telomere completeness (Li and Durbin 2024) for multiple species can resolve this question.

For the merged primary assembly, we identified 4,941 loci out of 5,310 Tetrapoda BUSCO loci (93.1%), of which 4,243 were single copy (79.9%), 370 were duplicated (6.97%), and 328 were fragmented (6.18%; Fig. 2a). For the transcriptome, we assembled 246,287 “genes” and 318,347 transcripts from the 2 tissues, with a contig N50 of 621 bp, a median contig length of 297 bp, and an average contig length 526 bp from 129 Mb of assembled data. Finally, we annotated mitochondrial genome assemblies, which match the existing reference (AY728227) for this species (Mueller *et al.* 2004) in terms of length, gene order, and base composition. They differ from each other by a 2 bp indel in the 16S ribosomal RNA gene, a single transition in cytochrome *b,* and 2 nearby indels in the putative control region/*D*-loop.

**Fig. 3.**
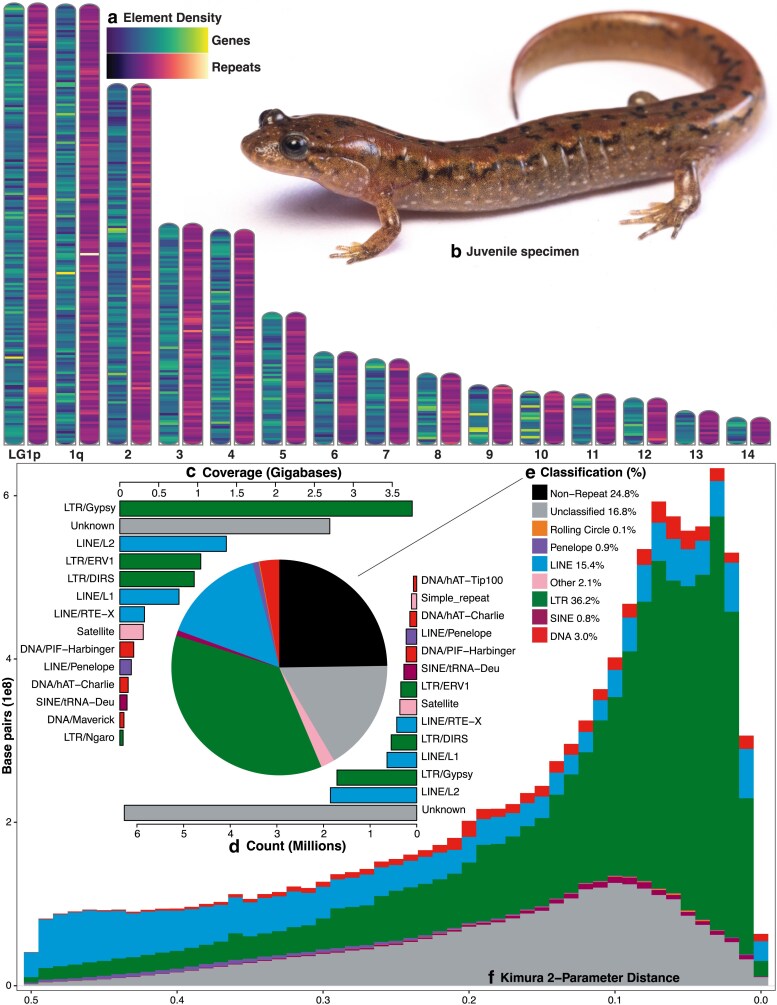
a) LG of the primary merged assembly mapped for gene and repeat density in 7.5 MB windows. b) A juvenile *Desmognathus fuscus “*B2” in life (photo courtesy of Max A. Seldes, GW). c to f) The transposable element landscape for aDesFus1-2.1 identified and annotated using the EarlGrey pipeline (Baril *et al.* 2024), showing coverage > 40 MB (c), TEs by count > 70 k (d), percentage by family (e), and relative K2P divergence (f). The divergence landscape (oldest to most recent, left to right) indicates an early proliferation and slow contraction of LINEs, followed by recent massive LTR expansion and smaller but substantial increases in unclassified elements and DNA transposons, with very little recent or non-divergent activity. “Other” includes simple, microsatellite, and RNA repeats.

For scaffolding, analysis of RNAseq data from a wild-type female and her 19 offspring yielded 208,631 SNPs that were heterozygous in the parent and therefore potentially informative for mapping. These were used to generate a framework map that incorporated 37,702 high-confidence SNPs that were highly replicated and presumably homozygous in all sires. Incorporation of lower confidence SNPs that were similar to high-confidence markers allowed us to incorporate another 94,185 informative SNPs that facilitated anchoring and orientation of assembled contigs. In total, linkage-mapping data scaffolded 4,286 contigs into 14 primary LG and 2 smaller scaffolds covering 7.5 Gb of the genome (Fig. 3a). This incorporates 47% of the assembly and 60% of annotated mRNAs (see below), and spans approximately half the predicted length of the genome based on densitometry estimates.

The longest 14 scaffolds range in length from 2.79 to 0.87 GB; we take these to represent portions of the 14 chromosomes. The 2 smaller scaffolds might be part of LG1 based on pairwise similarity in the linkage map, but we were unable to incorporate them directly. Synteny with *Ambystoma* is discernible using the 5,310 Tetrapoda BUSCO loci (Fig. 2c)—for instance, LG8 in *Desmognathus* appears to be partially homologous to chromosome 8 in *Ambystoma*. In contrast, most chromosomes and LG also show substantial rearrangements, apparently representing numerous fusions, fissions, and inversions over the ∼158 Ma separating Ambystomatidae and Plethodontidae (Stewart and Wiens 2025).

The level of synteny between *Ambystoma* and *Desmognathus* appears qualitatively lower than that of *Ambystoma* and *Pleurodeles* (Brown *et al.* 2025), despite a similarly deep divergence of ∼143 Ma (Stewart and Wiens 2025). We hypothesize that this difference might result from 3 primary mechanisms. First, it may reflect real differences in chromosome layouts, given dramatic reorganizations of physiology and genome structure in the group. Plethodontids differ from other salamanders in having lost their lungs (Ruben and Boucot 1989), transitioned to direct development (Wake and Hanken 1996), and in *Desmognathus,* reverted to a larval stage (Chippindale *et al.* 2004) and undergone a dramatic reduction in genome size (Liedtke *et al.* 2018). Any or all these factors may relate to genetic rearrangements—previous comparisons between *Ambystoma,* the frog *Xenopus,* and the bird *Gallus* revealed that apparently conserved amphibian synteny resulted from multiple independent lineage-specific fusions (Voss *et al.* 2011; Smith *et al.* 2019). Furthermore, early diverging salamander lineages such as Cryptobranchidae and Sirenidae have much larger and more variable karyotypes, including mixtures of macro- and micro-chromosomes (Voss *et al.* 2011), suggesting that complex processes may be at play across Urodela. Second, our scaffolding approach may have placed some LG out of order, perhaps owing to the genetic mismatch between the genomic and meiotic localities. Third, also related to scaffolding, more conserved syntenic blocks might be represented in the unplaced contigs. Future assemblies with more complete and accurate scaffolding can be used to test hypotheses regarding conservation of gene order across salamanders.

**Fig. 4.**
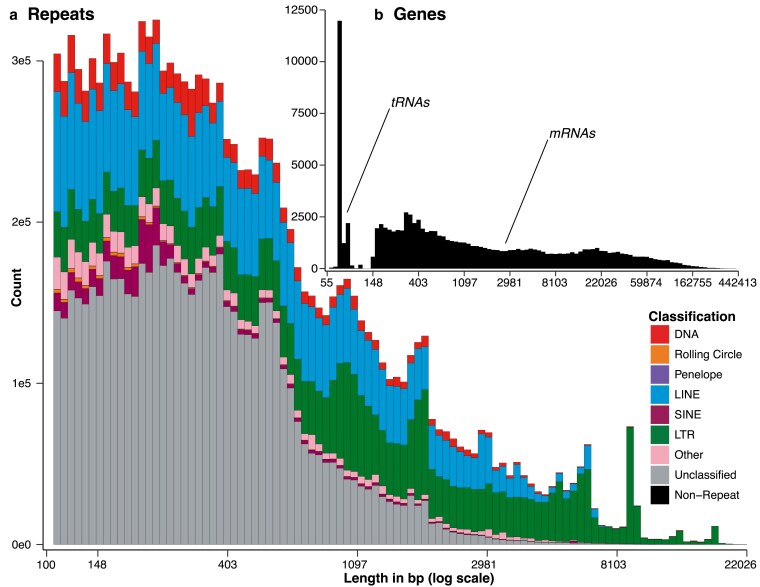
Length-frequency distributions for a) repeat elements (fig. 3), showing prevalence of shorter elements (e.g. DNA and SINE features) along with an extended tail of kilobase-scale LINE and LTR retrotransposons, and b) functional annotations, which clearly separate into a smaller mode of more prevalent tRNAs vs larger mRNAs at ∼150 bp in length.

Analysis of transposable elements in the primary assembly yielded several interesting patterns (Fig. 3). The relative divergence landscape in our assembly shows an early expansion and slow contraction of LINEs, followed by a quick recent expansion of DNA, LTR, and SINE elements (Fig. 3d). Similarly, unclassified elements expanded at roughly the same time, and very little of the genome consists of recent or non-divergent repeat activity. The median TE length is 351 bp with a mean of 878 bp and 95/99th percentiles of 3,571/8,858 bp, with LTRs forming several distinct modes of longer elements (Fig. 4a). The contribution of increased TE activity and low rates of DNA loss to genome expansion in salamanders is well known (Sun and Mueller 2014). How these dynamics promoted the decrease in *Desmognathus* genome size (Liedtke *et al.* 2018; Sclavi and Herrick 2019) represents a major question for future research.

Approximately 25% of the *Desmognathus* genome is single copy, while 75% is repetitive (Fig. 3e), greater than that of *Ambystoma* at 68% and similar to that of *Pleurodeles,* ranking among the highest percentages known in animals (Meyer *et al.* 2021; Schloissnig *et al.* 2021; Brown *et al.* 2025). However, *Desmognathus* is more similar to *Ambystoma* in having ∼36% of the genome representing LTRs and ∼15% representing LINEs, whereas the majority of *Pleurodeles* TEs are DNA-based, mostly hAT and Harbinger elements. Unlike *Ambystoma* and *Pleurodeles.* for which relatively few TEs were unknown, ∼17% of the *Desmognathus* TEs are unclassified (Fig. 3e). This may be an artifact of our partially scaffolded assembly or the identification steps in the EarlGrey pipeline, which we did not optimize for amphibians. There may also be novel elements or artifacts associated with *Desmognathus,* potentially related to the significant ancestral down-shift in genome size (Liedtke *et al.* 2018).

A novel finding in our analysis is a relatively high percentage of satDNA (∼2.5%) compared to *Ambystoma* and *Pleurodeles* (<0.5%); more similar to the ∼5% in *Homo* (Brown *et al.* 2025). Unlike *Ambystoma* and *Pleurodeles* where PIF/Harbinger elements are ∼7 to 20% of repeats by coverage, they are much less common (<3%) in *Desmognathus* and other plethodontids including some *Aneides, Batrachoseps, Eurycea,* and *Bolitoglossa* (Sun *et al.* 2012a, 2012b; Sun and Mueller 2014). Similar to earlier shotgun-based estimates for plethodontid and cryptobranchid salamanders (Sun *et al.* 2012a, 2012b; Sun and Mueller 2014) and the more complete recent genomes for ambystomatids and salamandrids (Schloissnig *et al.* 2021; Brown *et al.* 2025), LTR/Ty3 is the dominant TE in *Desmognathus,* comprising ∼3.8 GB or 31% of repeats by coverage and 12% by count (Fig. 3c to e). Others such as LINE/L2, LTR/DIRS, and LTR/ERV1 are similarly common across salamanders; by count, LINE/L2 elements are slightly more common (13%) than LTR/Ty3 in our assembly.

For the functional annotations, our results were slightly reduced in comparison to the recent *Pleurodeles* genome (Brown *et al.* 2025). We estimated 97,159 isoforms, of which 81,973 were mRNA, 15,881 were tRNA, and 26,536 were identified with a common name in at least 1 reference database. Of the latter, 12,408 were unique, which we take as a lower bound on the number of protein-coding genes in *Desmognathus,* compared to 18,799 in *Pleurodeles.* The average gene length is 9.4 kb, with an average exon length of 268 bp, an average protein length of 226aa. These length data are influenced by the significant mode of the much smaller tRNAs (Fig. 4); the median exon length is 664 bp and the 95/99 percentile is 52,819/120,797 bp. Other pipelines such as *BRAKER3* (Gabriel *et al.* 2024) may reveal additional annotations, but we did not explore these here, representing a substantial opportunity for future analyses.

We note that 1 previous study intentionally testing for a life-history/genome-size correlation in *Desmognathus* claimed to reject the hypothesis that terrestriality selects for smaller genomes, finding no relationship (Hally *et al.* 1986). However, the largest reported *C-*values of 18 to 22 pg (Bachmann 1970; Olmo 1974) were estimated for Desmognathus *“marmoratus”* and *Desmognathus “quadramaculatus”* (see Pyron and Beamer 2022, 2023b for recent taxonomic revisions of these complexes), which are both highly aquatic and lay their eggs in water, the ecological state associated with larger genomes in other salamander lineages (Lertzman-Lepofsky *et al.* 2019). Concomitantly, the smallest *C-*values (<14 pg) are associated with the terrestrial direct-developer *D. wrighti* (Hally *et al.* 1986; Sessions and Larson 1987). This and similar results for other terrestrial direct-developing Neotropical plethodontids recently discovered to have miniaturized genomes (Decena-Segarra *et al.* 2020) suggest the potential for complex interactions between natural history and genome size across Plethodontidae.

## Data Availability

The genome and transcriptome assemblies and raw reads for both HiFi (DNA) and Illumina (RNA) sequencing are deposited on SRA under BioProject PRJNA1038779 and BioSamples SAMN38201181-2 (SAMN40589320 for the merged reads) for the genome assembly and PRJNA1234120 for the RNAseq data from the mother + clutch for the linkage map. The mitochondrial genomes are deposited on GenBank under accessions OR794553/PP503028. This Whole Genome Shotgun project has been deposited at DDBJ/ENA/GenBank under the accessions JAXCVG000000000 (AMNH A-194068/Dfus1.0), JBBMEE000000000 (AMNH A-194069/Dfus2.0), and JBBULT000000000 (aDesFus1-2.1). The versions described in this paper are versions JAXCVG010000000, JBBMEE010000000, and JBBULT010000000, respectively. The transcriptome assemblies are available on GenBank under accessions GKTJ00000000 (AMNH A-194068 - liver) and GKTI00000000 (AMNH A-194069 - kidney). The scaffolded assembly in FASTA format (aDesFus1-2.1.fasta) and the TE (aDesFus1-2.1.filteredRepeats.gff) and functional (aDesFus1-2.1.gff3) annotations are provided in Zenodo repository https://doi.org/10.5281/zenodo.15200350.
